# 通过式固相萃取-超高效液相色谱-飞行时间质谱法快速筛查和确证水产品中36种磺胺类及4种四环素类药物

**DOI:** 10.3724/SP.J.1123.2024.12001

**Published:** 2025-08-08

**Authors:** Qianqian WANG, Zhongjie JU, Chengfei JIANG, Yan Zhang, Shuai WU, Jing LI, Kun CHU

**Affiliations:** 烟台市食品药品检验检测中心，山东 烟台 264000; Yantai Testing Center for Food and Drug，Yantai 264000，China

**Keywords:** 通过式固相萃取, 超高效液相色谱-飞行时间质谱, 磺胺类药物, 四环素类药物, 水产品, pass-through solid-phase extraction, ultra performance liquid chromatography-time-of-flight-mass spectrometry（UPLC-TOF-MS）, sulfonamide drugs, tetracycline drugs, aquatic products

## Abstract

为实现水产品中磺胺类及四环素类药物的快速、准确分析，本文将通过式固相萃取柱（PRiME HLB）与超高效液相色谱-飞行时间质谱（UPLC-TOF-MS）联用，建立了一种通用且能够快速筛查并确证水产品中36种磺胺类药物和4种四环素类药物多残留的分析方法。采用80%乙腈水溶液（含0.05 mol/L乙二胺四乙酸二钠）对样品进行提取，提取液经PRiME HLB固相萃取柱净化后氮吹至近干，用5%甲醇水溶液复溶，最后过聚四氟乙烯滤膜。以0.1%甲酸水溶液（含2 mmol/L乙酸铵溶液）和0.1%甲酸甲醇溶液为流动相进行梯度洗脱，采用飞行时间质谱的信息依赖采集（IDA）模式进行扫描，通过母离子精确*m/z*、保留时间、同位素丰度比和二级子离子谱库比对等信息实现目标物的快速筛查和确证，使用外标法（基质匹配混合标准溶液）定量。实验优化了UPLC的洗脱程序，能够在13 min内实现7组共17个同分异构体的良好分离。通过优化二级质谱的碰撞能量，获取全面的二级子离子质谱图，结合收集的化合物分子式、CAS号等信息，建立完善的数据库。此外，实验还比较了两种净化柱（PRiME HLB柱和HLB柱）的实验流程，优化了提取溶剂的组成及滤膜的种类，并获得了最佳实验条件。方法学验证结果表明，36种磺胺类和4种四环素类药物在2~50 μg/L范围内具有良好的线性关系，相关系数均≥0.990 68，除氨基磺胺和磺胺灭脓的定量限为10 μg/kg外，其他38种药物的定量限均为5 μg/kg。以草鱼肉、虾仁、扇贝丁和鲅鱼肉为空白基质样品，在3个加标水平（5、10、20 μg/kg）下，40种药物的回收率为62.8%~116.4%，相对标准偏差≤13.8%。该方法操作简便，稳定性好，通用性高，能够满足水产品中磺胺类及四环素类药物的快速筛查与确证需求。

## Abstract (en)

In order to achieve rapid and accurate analysis of sulfonamide and tetracycline drugs in aquatic products， a universal method for the rapid screening and confirmation of multiple residues of 36 sulfonamide and four tetracycline drugs was established. The method combined the PRiME HLB solid-phase extraction（SPE） column with ultra performance liquid chromatography-time-of-flight-mass spectrometry（UPLC-TOF-MS）. Samples were extracted using 80% acetonitrile aqueous solution（containing 0.05 mol/L Na_2_EDTA）. The resultant solution was purified by PRiME HLB SPE column and then blown to nearly dry with nitrogen. It was redissolved with 5% methanol aqueous solution and finally passed through a polytetrafluoroethylene filter membrane. Gradient elution was carried out using 0.1% formic acid aqueous solution（containing 2 mmol/L ammonium acetate solution） and 0.1% formic acid methanol solution as the mobile phases， and scanning was performed using the information dependent acquisition（IDA） mode of TOF-MS. The rapid screening and confirmation of the target compounds were achieved through information such as the precise *m/z* of the parent ion， retention time， isotope abundance ratio， and comparison with the secondary daughter-ion spectral library， and quantification is carried out by the external standard method（using the matrix-matched mixed standard solution）. The UPLC elution program was optimized to achieve good separation of 17 isomers in seven groups within 13 min. By optimizing the collision energy of the secondary mass spectrometry， a comprehensive secondary fragment ion mass spectrum is obtained. Combined with the collected information such as the chemical molecular formula and CAS number of the compounds， a complete database is established. In addition， the procedures of two purification columns（PRiME HLB column and HLB column） was compared， and the composition of the extraction solvent and the type of filter membrane were optimized to obtain the optimal experimental conditions. The methodological validation results indicated that 36 sulfonamide and four tetracycline drugs had a good linear relationship within the range of 2‒50 μg/L， and the correlation coefficients were all ≥0.990 68. Except for sulfanilamide and ambamide， whose LOQs were 10 μg/kg， the LOQs of the other 38 drugs were all 5 μg/kg. Using grass carp meat， shrimp meat， scallop meat and mackerel meat as blank matrix samples， at three spiked levels（5， 10 and 20 μg/kg）， the recoveries of the 40 drugs were 62.8%‒116.4%， and the RSDs were ≤13.8%. This method is simple to operate， has good stability and high universality， and can meet the requirements of rapid screening and confirmation of sulfonamide and tetracycline drugs in aquatic products.

磺胺类和四环素类药物均为人工合成的广谱抗生素，在临床应用中，常用于细菌性疾病的治疗与预防，同时也可用于寄生虫病的治疗^［1，2］^。鉴于这两类药物具备化学性质稳定、抗菌谱广泛、使用方便以及价格低廉等优点，它们在规模化养殖领域得到了广泛应用^［3，4］^。然而，此类药物稳定的化学性质致使它们在动物体内的代谢过程较为缓慢，进而容易在动物体内残留并蓄积。此外，养殖人员专业水平参差不齐，药物滥用现象时有发生，且休药期执行不规范，这最终会导致食品中药物残留量超标^［5］^。随着食物链的延伸，这些超标药物会在人体内逐渐累积。相关研究证实，过量摄入抗生素类药物可引发肾中毒^［6］^、过敏反应、抑制造血功能^［7，8］^，甚至存在致癌风险，对人类健康构成潜在威胁。因此，国外对磺胺类和四环素类药物一般都是限制使用^［9］^。国内近年来先后发布了《食品安全国家标准 食品中兽药最大残留限量》（GB 31650-2019^［10］^）和《食品安全国家标准 食品中41种兽药最大残留限量》（GB 31650.1-2022^［11］^）两项国家标准，对动物源食品中磺胺类药物的总残留限量和四环素类药物的残留限量做出了明确规定。其中，水产品中磺胺类药物的总残留限量规定：鱼（皮+肉）中的残留量不得超过100 µg/kg^［10］^；土霉素、金霉素、四环素3种四环素类药物的单个或组合残留限量规定：鱼（皮+肉）和虾（肌肉）中的残留量不得超过200 µg/kg^［10］^；多西环素（也是一种四环素类药物）的残留限量规定：鱼（皮+肉）中的残留量不得超过100 µg/kg^［10］^。随着人们生活水平的提升，高蛋白、高营养价值的水产品在饮食结构中的占比逐渐增加。在此背景下，磺胺类和四环素类药物的检测日益受到关注。如何快速、准确地检测这些药物，已成为亟待解决的关键问题之一。

目前，磺胺类和四环素类药物的检测方法主要有微生物法^［12，13］^、免疫检测法（包括酶联免疫吸附法和胶体金免疫层析法等）^［14，15］^、液相色谱法^［16，17］^及液相色谱-串联质谱法^［18‒20］^等。微生物法与免疫检测法操作简便，适用于大批量样本的快速筛查检测，但这两种方法存在稳定性差、准确度低的缺陷。液相色谱法的稳定性较好，但灵敏度低，检测通量小。液相色谱-串联质谱法的灵敏度高，抗干扰性强，现有的国家标准（如GB/T 21316-2007^［21］^、GB/T 21317-2007^［22］^、GB 31656.11-2021^［23］^等）多采用此法。上述国家标准方法基于串联四极杆低分辨质谱构建，可基本满足日常检验需求，但它们的前处理流程较为复杂，色谱分离耗时较长，检测通量小，且存在假阳性或假阴性结果的可能性，容易引发检验结果的误判。

水产品中磺胺类和四环素类药物的前处理方法有固相萃取法、分散固相萃取法、液液萃取法和QuEChERS法等^［24‒27］^，其中固相萃取法是应用较多的前处理方法。对于磺胺类和四环素类药物，HLB净化柱是最为常见的固相萃取柱^［24］^，但其操作步骤较为复杂且操作难度高，不适合批量样品的处理。PRiME HLB固相萃取柱属于通过式固相萃取柱，是近年来兴起的一种新型样品前处理净化技术。使用该净化柱时，无需进行活化、淋洗、洗脱等操作步骤，不仅操作简便，而且净化效率高，目前正广泛应用于兽药残留检测领域。

随着高分辨质谱技术的迅猛发展，飞行时间质谱凭借其高分辨率、高通量以及非靶向筛查等特性，在兽药残留筛查检测领域的应用日益广泛^［26，28］^。目前，针对水产品中磺胺类和四环素类药物（涉及多组同分异构体）的高通量筛查检测研究相对较少。通过查阅相关文献，尚未发现有关磺胺曲沙唑、磺胺甲氧吡嗪和磺胺氯吡嗪等17个同分异构体药物分离的研究报道。本研究通过优化色谱条件，在13 min内成功实现了7组共17个同分异构体药物的有效分离。通过减少仪器分析时间来缩短整体实验周期，提高了检测效率。本研究将通过式固相萃取柱与超高效液相色谱-飞行时间质谱（UPLC-TOF-MS）技术结合，对前处理过程进行了优化，确定了完整的实验流程；同时，结合数据库资源，建立了一种适用于水产品中36种磺胺类和4种四环素类药物的高通量、高效筛查与确证方法，旨在为保障人民食品安全提供有力支持。

## 1 实验部分

### 1.1 仪器、试剂与材料

LC-20AD超高效液相色谱仪（日本岛津公司）；X500R QTOF质谱仪（美国AB SCIEX公司）；BSA223S-CW分析天平（德国赛多利斯公司）；MS3 digital旋涡混匀器、Multi Reax振荡仪（德国IAK公司）；CR 18 RT冷冻离心机（上海天美生化仪器设备工程有限公司）；N-EVAP 112氮吹仪（美国ORGANOMATION公司）；JYL-C020E组织搅碎机（九阳股份有限公司）。

36种磺胺类药物标准品和4种四环素类药物标准品（质量浓度均为1 000 mg/L，溶剂均为乙腈）购自天津阿尔塔科技有限公司。实验用水为蒸馏水（广州屈臣氏食品饮料有限公司），甲醇、乙腈、甲酸、乙酸铵（色谱纯，德国Merck公司），乙二胺四乙酸二钠（Na_2_EDTA，分析纯）、乙酸（分析纯，国药集团化学试剂有限公司），正离子校正液（货号5049910，美国AB SCIEX公司）。Waters Oasis PRiME HLB固相萃取柱（6 mL/500 mg，美国Waters公司）；聚醚砜（PES）滤膜、尼龙（NYLON）滤膜、聚四氟乙烯（PTFE）滤膜（均为0.22 µm，安捷伦科技（中国）有限公司）。实际样品草鱼、虾仁、扇贝丁、鲅鱼均购自本地超市。

### 1.2 标准溶液的配制

#### 1.2.1 标准储备溶液的配制

分别准确移取1 mL上述40种标准品溶液于10 mL容量瓶中，用乙腈稀释、定容并摇匀，分别配制成质量浓度为100 mg/L的标准储备溶液，于‒20 ℃下冷冻保存。

#### 1.2.2 混合标准工作溶液和基质匹配混合标准溶液的配制

准确移取0.1 mL各标准储备溶液于10 mL容量瓶中，用乙腈稀释、定容并摇匀，配制成质量浓度为1 mg/L的混合标准中间溶液。临用前，用5%甲醇水溶液或空白基质提取液稀释混合标准中间溶液，配制成所需质量浓度的混合标准工作溶液或基质匹配混合标准溶液。

### 1.3 实验方法

#### 1.3.1 试样制备

取500 g样品（其中鱼类样品（含鱼皮）需先去头、去骨），用组织搅碎机充分捣碎、混匀，得到实验用鱼肉（包含草鱼和鲅鱼（含皮））、虾仁和扇贝丁样品，于‒18 ℃下冷冻保存。

#### 1.3.2 样品前处理

将样品解冻至室温，准确称取2.00 g（±0.05 g）样品，加入8 mL 80%乙腈水溶液（含0.05 mol/L Na_2_EDTA），涡旋混匀后振荡提取20 min，在5 000 r/min下离心5 min。取5 mL上清液过PRiME HLB固相萃取柱（无需活化），弃去前0.5 mL流出液，收集后续4 mL流出液，氮吹至近干，用1 mL 5%甲醇水溶液复溶，过0.22 µm PTFE滤膜，上机分析。

### 1.4 分析条件

#### 1.4.1 色谱条件

色谱柱：Waters ACQUITY UPLC BEH C18 柱（100 mm×2.1 mm，1.7 µm）；柱温：40 ℃；进样量：5 µL；流速：0.35 mL/min。流动相A为0.1%甲酸水溶液（含2 mmol/L乙酸铵溶液），B为0.1%甲酸甲醇。梯度洗脱程序：0~0.5 min，90%A；0.5~5 min，90%A~80%A；5~7 min，80%A~60%A；7~8.5 min，60%A；8.5~9.5 min，60%A~10%A；9.5~11.5 min，10%A；11.5~11.6 min，10%A~90%A；11.6~13 min，90%A。

#### 1.4.2 质谱条件

离子源：ESI源，正离子模式；喷雾电压（IS）：5 500 V；雾化气压力（GS1）：0.345 MPa；辅助气压力（GS2）：0.379 MPa；气帘气压力（CUR）：0.241 MPa；离子源温度（TEM）：550 ℃；数据采集模式：信息依赖采集（IDA）模式。一级质谱参数：扫描范围*m/z* 50~650，累计时间0.1 s，去簇电压50 V，碰撞能量10 eV。二级质谱参数：扫描范围*m/z* 50~650，累计时间0.1 s，去簇电压50 V，碰撞能量35 eV，碰撞能量的变化步阶15 eV。触发二级扫描的响应阈值：10 cps；IDA触发：10个子离子扫描。在实验过程中，每完成5针样品分析后，使用与仪器匹配的正离子校正液，通过色谱数据系统（CDS）自动对仪器执行一次质谱模式及联用质谱模式的质量精度校正。36种磺胺类和4种四环素类药物的质谱参数和保留时间等信息见表1。

**表1:** 36种磺胺类和4种四环素类药物的质谱参数和保留时间

Compound	CAS No.	Molecular formula	*t*_R_/min	Precursor ion（*m/z*）	Fragment ion（*m/z*）
Sulfonamide drugs					
Ambamide（磺胺灭脓）	138-39-6	C_14_H_18_N_4_O_2_	0.81	187.05358^*^	170.0276
Sulfaguanidine（磺胺脒）	57-67-0	C_7_H_10_N_4_O_2_S	1.04	215.05972^*^	108.0445
Sulfanilamide（氨基磺胺）	63-74-1	C_6_H_8_N_2_O_2_S	1.12	173.03793^*^	108.0446
Sulfacetamide（磺胺醋酰）	144-80-9	C_8_H_10_N_2_O_3_S	1.94	215.04849^*^	108.0444
Sulfadiazine（磺胺嘧啶）	68-35-9	C_10_H_10_N_4_O_2_S	2.63	251.05972^*^	156.0116
Sulfisomidine（磺胺二甲异嘧啶）	515-64-0	C_12_H_14_N_4_O_2_S	2.86	279.09102^*^	124.0868
Sulfathiazole（磺胺噻唑）	72-14-0	C_9_H_9_N_3_O_2_S_2_	3.28	256.02090^*^	156.0117
Sulfapyridine（磺胺吡啶）	144-83-2	C_11_H_11_N_3_O_2_S	3.67	250.06447^*^	156.0115
Sulfamerazine（磺胺甲基嘧啶）	127-79-7	C_11_H_12_N_4_O_2_S	4.07	265.07537^*^	156.0116
Diaveridine（二甲氧苄氨嘧啶）	5355-16-8	C_7_H_10_N_2_O_2_S	5.18	261.13460^*^	245.1028
Dapsone（氨苯砜）	80-08-0	C_12_H_12_N_2_O_2_S	5.25	249.06923^*^	156.0115
Sulfameter（磺胺对甲氧嘧啶）	651-06-9	C_11_H_12_N_4_O_3_S	5.26	281.07029^*^	156.0119
Sulfamoxole（磺胺噁唑）	729-99-7	C_11_H_13_N_3_O_3_S	5.56	268.07504^*^	156.0113
Sulfamethizole（磺胺甲噻二唑）	144-82-1	C_9_H_10_N_4_O_2_S_2_	5.63	271.03179^*^	156.0114
Sulfamethazine（磺胺二甲基嘧啶）	57-68-1	C_12_H_14_N_4_O_2_S	5.73	279.09102^*^	92.0495
Trimethoprim（甲氧苄氨嘧啶）	738-70-5	C_14_H_18_N_4_O_3_	5.99	291.14517^*^	230.1161
Sulfamethoxypyridazine（磺胺甲氧哒嗪）	80-35-3	C_11_H_12_N_4_O_3_S	6.28	281.07029^*^	156.0114
Sulfamonomethoxine（磺胺间甲氧嘧啶）	1220-83-3	C_11_H_12_N_4_O_3_S	6.66	281.07029^*^	156.0114
Succinylsulfathiazole（丁二酰磺胺噻唑）	116-43-8	C_13_H_13_N_3_O_5_S_2_	6.69	356.03694^*^	256.0281
Sulfachloropyridazine（磺胺氯哒嗪）	80-32-0	C_10_H_9_ClN_4_O_2_S	6.73	285.02075^*^	156.0115
Sulfamethoxazole（磺胺甲噁唑）	729-99-7	C_11_H_13_N_3_O_3_S	7.06	254.05939^*^	156.0116
Ormetoprim（二甲氧甲基苄氨嘧啶）	6981-18-6	C_13_H_16_N_4_O_2_	7.07	275.15025^*^	259.1187
Sulfamethoxypyrazine（磺胺甲氧吡嗪）	152-47-6	C_11_H_12_N_4_O_3_S	7.15	281.07029^*^	108.0445
Sulfatroxazole（磺胺曲沙唑）	23256-23-7	C_11_H_13_N_3_O_3_S	7.24	268.07504^*^	156.0115
Sulfadoxine（磺胺邻二甲氧嘧啶）	2447-57-6	C_12_H_14_N_4_O_4_S	7.54	311.08085^*^	156.0115
Sulfisoxazole（磺胺异噁唑）	127-69-5	C_11_H_13_N_3_O_3_S	7.63	268.07504^*^	156.0113
Sulfabenzamide（苯甲酰磺胺）	127-71-9	C_13_H_12_N_2_O_3_S	7.93	277.06414^*^	156.0115
Sulfaethoxypyridazine（磺胺乙氧基哒嗪）	963-14-4	C_12_H_14_N_4_O_3_S	8.22	295.08594^*^	156.0118
Sulfaphenazole（磺胺苯吡唑）	526-08-9	C_15_H_14_N_4_O_2_S	8.38	315.09102^*^	156.0118
Sulfaclozine（磺胺氯吡嗪）	102-65-8	C_10_H_9_ClN_4_O_2_S	8.40	285.02075^*^	108.0449
Sulfadimethoxine（磺胺间二甲氧嘧啶）	122-11-2	C_12_H_14_N_4_O_4_S	8.82	311.08085^*^	156.0114
Sulfabenz（磺胺苯）	127-77-5	C_12_H_12_N_2_O_2_S	8.84	249.06923^*^	108.0448
Sulfapyrazole（磺胺吡唑）	852-19-7	C_16_H_16_N_4_O_2_S	9.08	329.10667^*^	172.0869
Sulfaquinoxaline（磺胺喹噁啉）	59-40-5	C_14_H_12_N_4_O_2_S	9.18	301.07537^*^	156.0116
Sulfanitran（磺胺硝苯）	122-16-7	C_14_H_13_N_3_O_5_S	10.32	336.06487^*^	294.0560
Sulfasalazine（柳氮磺吡啶）	599-79-1	C_18_H_14_N_4_O_5_S	10.54	399.07577^*^	381.0649
Tetracycline drugs					
Tetracycline（四环素）	60-54-8	C_22_H_24_N_2_O_8_	6.81	445.16054^*^	410.1242
Oxytetracycline（土霉素）	79-57-2	C_22_H_24_N_2_O_9_	7.02	461.15546^*^	426.1188
Chlortetracycline（金霉素）	57-62-5	C_22_H_23_ClN_2_O_8_	8.55	479.12157^*^	444.0850
Doxycycline（多西环素）	564-25-0	C_22_H_24_N_2_O_8_	10.06	445.16054^*^	428.1339

* Quantitative ion.

### 1.5 数据处理

样品进样、数据采集和数据处理均由SCIEX OS软件（美国AB SCIEX公司）实现，数据库的构建基于Library View软件（美国AB SCIEX公司）实现。数据处理参数设置如下：精确母离子*m/z*偏差设为5×10^‒6^，占比为30%；保留时间偏差设为±5%，占比为25%；同位素丰度比差异设为±15%，占比为15%；二级碎片质谱库匹配得分大于70分，占比为30%。

## 2 结果与讨论

### 2.1 色谱条件的优化

由于同分异构体的精确母离子*m/z*完全一致，无法通过质谱条件实现定性和定量分析，因此液相色谱条件的优化显得尤为重要。对于难分离的同分异构体，通常需采用特殊性能色谱柱或延长液相洗脱时间以实现完全分离，但这不仅增加了实验成本，还延长了分析周期。

本研究中的磺胺类和四环素类药物共涉及7组（z1~z7）17种同分异构体，具体包括：磺胺二甲异嘧啶与磺胺二甲基嘧啶，磺胺噁唑、磺胺曲沙唑与磺胺异噁唑，磺胺对甲氧嘧啶、磺胺甲氧哒嗪、磺胺间甲氧嘧啶与磺胺甲氧吡嗪，磺胺邻二甲氧嘧啶与磺胺间二甲氧嘧啶，磺胺氯哒嗪与磺胺氯吡嗪，氨苯砜与磺胺苯，四环素与多西环素。其中，磺胺甲氧哒嗪、磺胺对甲氧嘧啶、磺胺间甲氧嘧啶与磺胺甲氧吡嗪4种同分异构体因保留时间相近，采用常规梯度洗脱程序难以实现分离。本文采用ACQUITY UPLC BEH C18色谱柱（100 mm×2.1 mm，1.7 µm），以0.1%甲酸水溶液（含2 mmol/L乙酸铵）和0.1%甲酸甲醇溶液为流动相，通过逐步优化色谱洗脱程序，在13 min内实现了所有同分异构体的基线分离（分离结果见图1）。与《动物源性食品中磺胺类药物残留量的测定 高效液相色谱-质谱/质谱法》（GB/T 21316-2007^［21］^）相比，本方法的分析时间缩短了27 min，且同分异构体分析数量由7种扩展至17种，有效提高了检测效率。

**图1:**
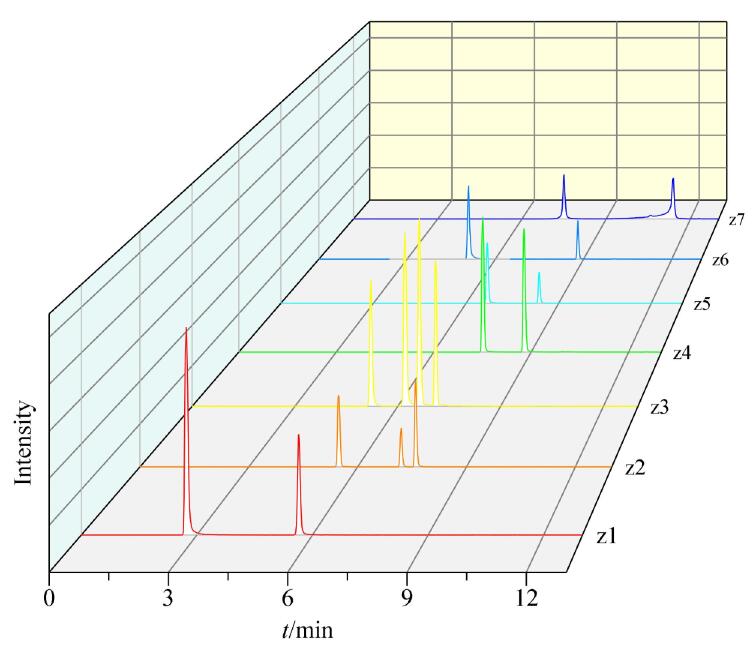
最佳色谱条件下7组（z1~z7）同分异构体的色谱图

### 2.2 质谱条件优化和数据库的建立

X500R QTOF质谱仪通过CDS实现质量精度的自动校正，从而确保仪器数据的稳定性。该仪器配备动态背景扣除功能，可极大地降低不同基质背景信号的干扰，增强样品中待测目标物的二级质谱信号强度。为获取高质量的高分辨质谱图，本研究通过扩展碰撞能量范围，分别采集高质量数与低质量数区域的质谱碎片信息，进而构建每种化合物的精准二级质谱库。经实验优化，二级质谱的碰撞能量参数最终设定为基础碰撞能量35 eV，碰撞能量步阶15 eV。在此条件下，每种化合物均可获得由（35±15） eV范围内不同碰撞能量叠加生成的二级质谱图。将质谱图数据导入Library View数据库软件，结合化合物的分子式、CAS号等信息，构建完善的化合物数据库。

配制质量浓度为200 μg/L的混合标准工作溶液，在优化后的仪器条件下完成数据采集，得到包含精确母离子*m/z*、二级质谱图和保留时间等信息的数据。随后，将这些数据导入Library View软件，建立相应的数据库。最佳仪器条件下40种目标化合物的提取离子色谱图见图2。以磺胺甲噁唑为例，标准谱库中磺胺甲噁唑的色谱图与二级质谱图见附图1（www.chrom-China.com）。

**图2:**
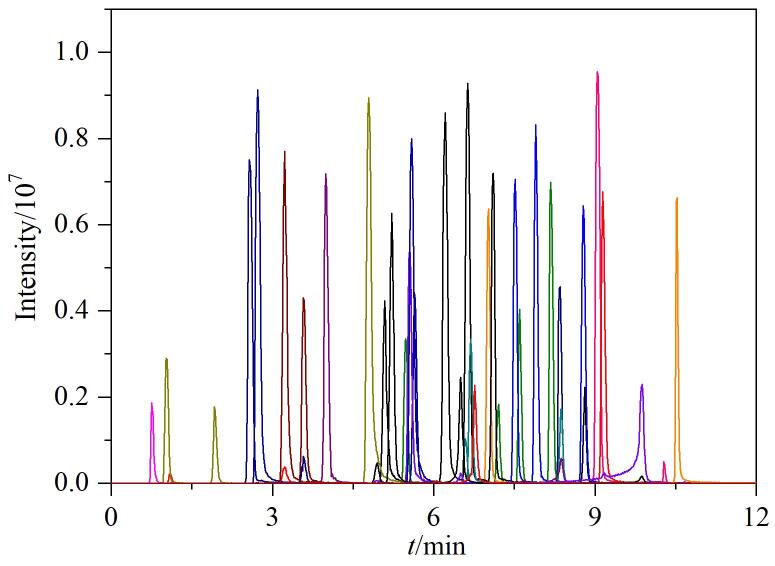
40种目标化合物混合标准工作溶液（200 μg/L）的提取离子色谱图

### 2.3 前处理条件的优化

#### 2.3.1 净化柱的选择

鱼、虾、贝类等水产品中富含磷脂、蛋白质等物质，这些物质会干扰目标化合物的分析，导致回收率降低，同时还可能污染仪器，增加仪器的维护成本。因此，在对样品进行提取后，通常需要对提取液进行净化处理，再进行仪器分析。HLB净化柱是常用的净化材料，其前处理流程如下：先对样品进行提取，接着离心，然后活化净化柱（约需15 min），再将提取液上样至净化柱中进行净化（约20 min），之后淋洗净化柱（约30 min），再进行洗脱（约15 min），最后进行氮吹、复溶操作，过滤膜后即可上机分析。

本文采用PRiME HLB通过式固相萃取净化柱。PRiME HLB是一种具有水可浸润性的强亲水性聚合物，具备独特的亲水-疏水平衡特性。它能够将磷脂等杂质吸附在净化柱内，而目标化合物则随提取液流出柱体。PRiME HLB固相萃取净化柱的前处理流程较为简便，具体如下：在对样品进行提取和离心后，直接将提取液上样至净化柱中净化（约20 min），随后进行氮吹和复溶操作，过滤膜后即可上机分析。与传统的HLB净化柱相比，PRiME HLB净化柱无需活化、淋洗和洗脱等步骤，直接收集过柱后的净化液即可开展后续实验，这使得实验时间缩短了约60 min，能够极大地提高检测效率。此外，通过使用该固相萃取柱进行净化处理，也获得了满意的回收率结果（详见2.4.3节）。因此，本研究选择PRiME HLB固相萃取柱进行净化实验。

#### 2.3.2 提取溶剂的选择

本研究以草鱼肉为样品基质，分别以36种磺胺类和4种四环素类药物的平均回收率为指标，考察了乙腈水溶液中不同体积分数（60%、70%、80%、90%、100%）的乙腈对两类药物的提取效果，结果见图3。由图3可以看出，当乙腈的体积分数为80%时，两类药物的回收率达到最高，提取效果最为理想。分析其原因，当乙腈比例较低时，溶液中水的比例相对较高，会提取出更多的杂质，这些杂质会对目标化合物的响应产生较大的基底干扰，进而影响目标物的准确检测；而当乙腈比例过高时，由于乙腈具有沉淀蛋白质的作用，对于高蛋白含量的鱼肉样本，会出现鱼肉聚团的现象，从而导致目标物提取不充分。

**图3:**
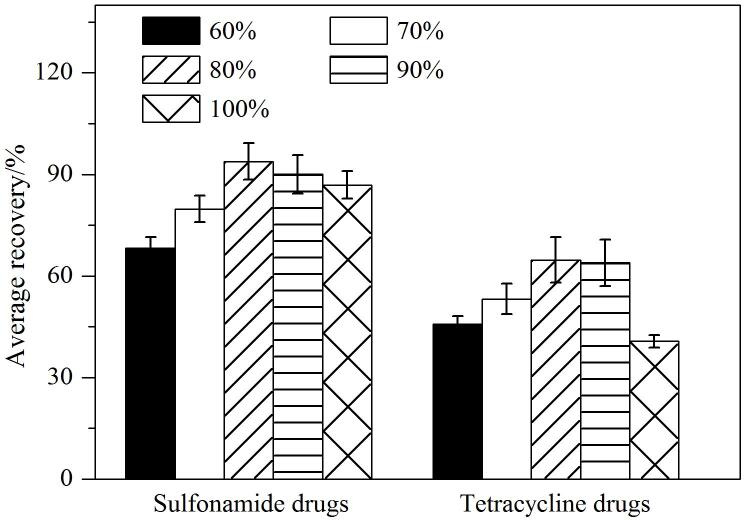
不同体积分数的乙腈对磺胺类药物和四环素类药物平均回收率的影响（*n*=3）

从图3中可以看出，四环素类药物的回收率整体偏低。原因是在提取过程中，四环素类化合物会与基质中的金属离子发生络合反应^［29］^，进而导致其提取不充分。因此，本文选择在80%乙腈水溶液中添加一定浓度的Na_2_EDTA，以此来提高四环素类化合物的回收率。图4展示了在80%乙腈水溶液中添加不同浓度的Na_2_EDTA对四环素类药物回收率的影响。从图中可以看出，随着Na_2_EDTA添加浓度的逐渐增大，四环素类药物的回收率也逐步提高。当Na_2_EDTA的浓度增大到0.05 mol/L之后，回收率趋于稳定。这说明0.05 mol/L的Na_2_EDTA已经能够充分占据提取液中的金属离子位点，从而将四环素类化合物从络合态中释放出来，因此无需再继续增加Na_2_EDTA的浓度。综上，本研究最终选用80%乙腈水溶液（含0.05 mol/L Na_2_EDTA）作为提取溶剂。

**图4:**
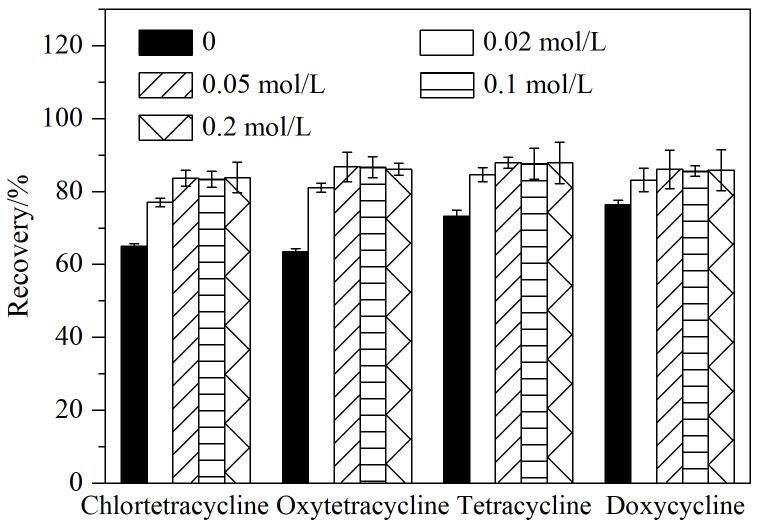
不同浓度的Na_2_EDTA对4种四环素类药物回收率的影响（*n*=3）

#### 2.3.3 滤膜的选择

不同滤膜对各类化合物的过滤和吸附效果存在差异，可能会导致目标化合物的损失，进而对回收率产生较大影响，降低检测结果的准确度。本文选取实验室常用的3种滤膜（材质分别为PES、NYLON和PTFE），以40种目标化合物的损失率（（过滤前目标化合物含量‒过滤后目标化合物含量）/过滤前化合物含量×100%）为考察指标，比较了不同滤膜对36种磺胺类和4种四环素类药物的过滤效果。结果表明，经PES滤膜过滤后，磺胺喹噁啉和磺胺灭脓的损失率超过10%；磺胺硝苯、金霉素和多西环素的损失率超过30%；甲氧苄氨嘧啶、二甲氧苄氨嘧啶和二甲氧甲基苄氨嘧啶的损失率更是超过80%。这表明PES滤膜对这些药物具有较强的吸附性，导致目标化合物损失较大。经NYLON滤膜过滤后，磺胺间二甲氧嘧啶、磺胺氯哒嗪、磺胺氯吡嗪、氨苯砜、磺胺苯、磺胺甲噻二唑、苯甲酰磺胺、磺胺喹噁啉、磺胺苯吡唑、丁二酰磺胺噻唑和磺胺吡唑的损失率超过10%；磺胺硝苯和柳氮磺吡啶的损失率超过90%。这说明NYLON滤膜对这些药物均具有较强的吸附作用，同样不适合用于这些药物的过滤。经PTFE滤膜过滤后，36种磺胺类和4种四环素类药物的损失率均小于10%，这表明PTFE滤膜对这40种目标化合物基本无吸附。因此，本研究最终使用0.22 µm PTFE滤膜进行过滤。

### 2.4 方法学验证

#### 2.4.1 基质效应

按1.2节方法分别配制溶剂混合标准工作溶液和3种样品（草鱼肉、虾仁、扇贝丁）基质匹配混合标准溶液，并分别绘制相应的标准曲线。采用相对响应值法来考察基质效应（ME），即ME=基质匹配标准曲线的斜率/溶剂标准曲线的斜率^［30］^。当ME˂100%时，表示存在基质抑制效应；当ME˃100%时，表示存在基质增强效应；当ME值为80%~120%时，基质效应可以忽略。实验结果表明，丁二酰磺胺噻唑在虾仁和扇贝丁中呈现出基质增强效应，在草鱼肉中呈现出基质抑制效应，而其他35种磺胺类药物在3种水产品样品中均表现为基质抑制效应；多西环素在草鱼肉和虾仁中呈现出基质抑制效应，在扇贝丁样品中呈现出基质增强效应，而其他3种四环素类药物在3种水产品样品中均表现为基质增强效应（见附表1）。由此可见，这两类药物在不同基质中的基质效应差异较大，且均不可忽略。因此，为降低基质效应的影响并提高结果的准确度，本方法采用基质匹配混合标准溶液进行后续定量分析。

#### 2.4.2 线性范围、检出限和定量限

取4种空白样品（草鱼肉、虾仁、扇贝丁、鲅鱼肉），按1.3节方法进行试样制备和前处理，得到空白基质提取液，再按照1.2.2节方法配制系列质量浓度（2、5、10、20、50 μg/L）的基质匹配混合标准溶液，按1.4节条件进样分析。以目标化合物定量母离子的峰面积为纵坐标（*y*），质量浓度为横坐标（*x*， μg/L），绘制基质匹配标准曲线。实验数据表明，40种目标化合物在2~50 μg/L范围内线性关系良好，相关系数（*r* ^2^）均≥0.990 68。分别以3倍信噪比（*S/N*）和10倍*S/N*计算检出限（LOD）和定量限（LOQ），结果表明，除两种磺胺类药物（氨基磺胺和磺胺灭脓）的LOD为4 μg/kg、LOQ为10 μg/kg外，其余34种磺胺类药物和4种四环素类药物的LOD均为2 μg/kg、LOQ均为5 μg/kg。以空白草鱼肉样品为例，相关数据结果见表2，其他3种空白样品（虾仁、扇贝丁、鲅鱼肉）的数据结果见附表2。上述实验结果表明，本方法具有较高的灵敏度。

**表2:** 36种磺胺类和4种四环素类药物的线性范围、线性方程、相关系数、检出限和定量限

Compound	Linear range/（μg/L）	Linear equation	*r* ^2^	LOD/（μg/kg）	LOQ/（μg/kg）
Sulfonamide drugs					
Ambamide	2‒50	*y*=682.6*x‒*873.9	0.99752	4	10
Sulfaguanidine	2‒50	*y*=6249.7*x*+15732.9	0.99258	2	5
Sulfanilamide	2‒50	*y*=624.2*x*+417.4	0.99551	4	10
Sulfacetamide	2‒50	*y*=3332.5*x*+10498.3	0.99327	2	5
Sulfadiazine	2‒50	*y*=10221.3*x*+9601.4	0.99703	2	5
Sulfisomidine	2‒50	*y*=17108.6*x*+36711.4	0.99621	2	5
Sulfathiazole	2‒50	*y*=7139.4*x*+9848.6	0.99420	2	5
Sulfapyridine	2‒50	*y*=13354.1*x*+28016.5	0.99447	2	5
Sulfamerazine	2‒50	*y*=14308.3*x*+14916.8	0.99362	2	5
Diaveridine	2‒50	*y*=22863.1*x*+22903.8	0.99824	2	5
Dapsone	2‒50	*y*=17420.1*x*+13277.5	0.99068	2	5
Sulfameter	2‒50	*y*=14183.4*x*+10873.4	0.99435	2	5
Sulfamoxole	2‒50	*y*=12374.0*x*+14861.1	0.99150	2	5
Sulfamethizole	2‒50	*y*=6755.9*x*+8634.3	0.99429	2	5
Sulfamethazine	2‒50	*y*=17003.6*x*+61230.6	0.99166	2	5
Trimethoprim	2‒50	*y*=44349.7*x*+51203.8	0.99413	2	5
Sulfamethoxypyridazine	2‒50	*y*=18869.3*x*+4990.4	0.99854	2	5
Sulfamonomethoxine	2‒50	*y*=12809.6*x*+50416.1	0.99088	2	5
Succinylsulfathiazole	2‒50	*y*=2302.9*x‒*612.1	0.99684	2	5
Sulfachloropyridazine	2‒50	*y*=6984.7*x*+12717.2	0.99103	2	5
Sulfamethoxazole	2‒50	*y*=15187.8*x*+16357.2	0.99538	2	5
Ormetoprim	2‒50	*y*=29060.0*x*+25721.8	0.99802	2	5
Sulfamethoxypyrazine	2‒50	*y*=18823.2*x*+8951.0	0.99578	2	5
Sulfatroxazole	2‒50	*y*=15582.4*x*+65771.2	0.99941	2	5
Sulfadoxine	2‒50	*y*=29094.8*x*+34826.9	0.99705	2	5
Sulfisoxazole	2‒50	*y*=10712.1*x*+33089.3	0.99364	2	5
Sulfabenzamide	2‒50	*y*=13019.0*x*+20705.7	0.99663	2	5
Sulfaethoxypyridazine	2‒50	*y*=19268.2*x*+32275.3	0.99923	2	5
Sulfaphenazole	2‒50	*y*=21574.6*x*+31691.0	0.99801	2	5
Sulfaclozine	2‒50	*y*=5792.8*x*+7352.3	0.99396	2	5
Sulfadimethoxine	2‒50	*y*=27287.2*x*+75266.0	0.99132	2	5
Sulfabenz	2‒50	*y*=2735.3*x*+2311.4	0.99569	2	5
Sulfapyrazole	2‒50	*y*=33284.9*x*+38752.3	0.99587	2	5
Sulfaquinoxaline	2‒50	*y*=14140.6*x*+19985.4	0.99319	2	5
Sulfanitran	2‒50	*y*=1843.9*x*+1.6	0.99652	2	5
Sulfasalazine	2‒50	*y*=5538.2*x*+2939.4	0.99958	2	5
Tetracycline drugs					
Tetracycline	2‒50	*y*=3048.9*x‒*358.0	0.99942	2	5
Oxytetracycline	2‒50	*y*=1955.6*x*+151.8	0.99507	2	5
Chlortetracycline	2‒50	*y*=1630.9*x*+1659.9	0.99602	2	5
Doxycycline	2‒50	*y*=2474.5*x*+2322.8	0.99625	2	5

*y*： peak area； *x*： mass concentration， μg/L.

#### 2.4.3 回收率和精密度

在4种空白样品（草鱼肉、白虾仁、扇贝丁、鲅鱼肉）基质中分别添加低、中、高3个水平（5、10、20 μg/kg）的混合标准工作溶液，进行加标回收试验，每个加标水平制备6个平行样，计算回收率和相对标准偏差（RSD）。虾仁、扇贝丁和鲅鱼肉样品基质中的实验结果见表3，草鱼肉样品基质中的实验结果见附表3。结果显示，在3个加标水平下，36种磺胺类和4种四环素类药物在4种样品基质中的加标回收率分别为63.4%~116.4%和62.8%~96.0%，RSD均≤13.8%。其中，氨基磺胺和磺胺灭脓的LOQ为10 μg/kg，因此在5 μg/kg加标水平下无法计算二者的回收率和RSD。在上述结果说明本方法的回收率和精密度均较高，通用性强，能够满足水产品中36种磺胺类和4种四环素类药物的快速筛查与确证要求。

**表3:** 3种空白样品（扇贝丁、虾仁和鲅鱼肉）基质中36种磺胺类和4种四环素类药物的加标回收率和相对标准偏差（*n*=6）

Compound	Scallop meat						Shrimp meat						Mackerel meat					
	5 μg/kg		10 μg/kg		20 μg/kg		5 μg/kg		10 μg/kg		20 μg/kg		5 μg/kg		10 μg/kg		20 μg/kg	
	Rec.	RSD	Rec.	RSD	Rec.	RSD	Rec.	RSD	Rec.	RSD	Rec.	RSD	Rec.	RSD	Rec.	RSD	Rec.	RSD
Sulfonamide drugs																		
Ambamide	/	/	96.0	5.5	101.5	6.4	/	/	83.0	6.8	72.2	4.3	/	/	109.5	4.9	109.9	8.2
Sulfaguanidine	78.8	8.9	101.3	2.9	92.6	11.5	80.5	4.7	89.2	7.2	75.5	5.3	85.1	4.7	90.8	4.1	88.1	2.7
Sulfanilamide	/	/	107.8	3.2	102.6	13.1	/	/	108.7	13.1	90.4	12.0	/	/	80.1	9.7	81.8	9.9
Sulfacetamide	86.9	5.2	96.1	4.5	103.5	12.6	74.4	1.1	86.8	9.6	71.7	5.4	81.7	4.7	82.0	3.2	83.9	2.2
Sulfadiazine	92.3	5.4	93.5	2.9	93.8	7.9	87.0	1.7	102.2	9.1	86.1	4.0	86.0	4.3	88.5	4.3	86.8	2.2
Sulfisomidine	88.7	1.6	91.3	3.5	87.6	9.1	82.9	2.9	98.2	8.9	83.9	5.2	78.3	5.3	81.1	4.5	81.3	3.8
Sulfathiazole	97.1	2.5	98.4	4.4	93.1	10.6	78.8	4.4	85.9	10.3	76.6	4.6	72.2	5.7	74.1	4.1	75.8	5.0
Sulfapyridine	92.9	2.0	91.9	3.7	86.8	7.4	86.2	2.0	101.0	4.9	88.9	3.5	86.4	4.7	88.5	3.4	85.4	1.8
Sulfamerazine	91.8	2.9	91.6	4.8	89.7	8.4	89.1	1.5	103.3	10.0	87.4	3.9	80.8	4.0	83.6	3.9	80.9	2.0
Diaveridine	89.9	4.5	91.1	3.9	90.5	8.1	79.5	2.3	89.7	6.8	76.5	1.1	69.0	6.6	74.4	9.0	74.7	1.6
Dapsone	83.6	4.7	92.3	8.8	89.3	6.3	91.8	1.6	105.7	5.3	90.2	3.5	80.9	3.8	84.2	3.9	82.1	1.5
Sulfameter	94.7	2.5	94.8	6.0	91.8	7.8	96.8	2.0	112.3	8.4	96.4	2.6	81.8	3.6	85.3	4.7	84.4	1.5
Sulfamoxole	112.5	5.7	110.1	3.7	105.5	7.1	96.1	2.8	112.8	6.3	97.8	3.8	82.1	4.3	85.4	3.2	83.1	1.9
Sulfamethizole	87.1	2.3	90.0	3.3	86.2	7.6	92.9	2.7	109.2	8.9	89.3	4.2	78.4	4.3	80.5	3.7	77.5	1.2
Sulfamethazine	96.6	2.7	95.2	3.3	92.3	5.5	91.4	2.1	101.3	8.3	84.7	3.7	86.2	5.1	89.1	3.8	87.2	2.0
Trimethoprim	91.5	4.5	93.0	3.9	92.2	8.2	81.4	2.3	93.9	5.8	80.0	2.6	71.3	6.6	75.2	6.8	76.3	1.3
Sulfamethoxypyridazine	92.9	3.4	92.1	3.7	84.1	6.3	88.8	2.0	99.0	8.9	83.3	2.7	84.8	4.1	88.2	4.8	85.7	1.8
Sulfamonomethoxine	88.7	2.6	89.0	3.2	87.1	4.8	77.1	3.6	93.6	9.1	82.1	4.4	76.5	3.9	78.6	4.0	74.9	3.1
Succinylsulfathiazole	77.4	6.3	86.3	4.5	79.0	9.7	76.4	4.8	94.6	10.1	82.3	3.0	79.9	6.3	80.2	5.9	78.8	5.5
Sulfachloropyridazine	80.7	2.7	85.2	3.7	82.4	8.4	79.6	3.5	98.0	7.8	85.2	4.0	76.5	4.6	78.3	4.4	76.2	3.4
Sulfamethoxazole	98.3	3.0	91.2	6.0	88.0	9.4	86.4	4.9	98.0	10.2	79.4	5.2	78.4	4.9	81.9	3.7	77.7	2.8
Ormetoprim	91.5	1.5	91.9	3.4	89.0	7.8	74.7	2.8	83.8	6.9	72.1	2.5	63.4	8.6	69.0	9.1	67.5	3.4
Sulfamethoxypyrazine	85.8	2.3	86.4	6.1	82.3	7.5	78.0	5.7	96.2	8.4	82.4	4.9	87.5	4.0	92.0	3.5	89.4	2.4
Sulfatroxazole	100.3	1.5	95.8	4.2	92.8	8.4	76.5	5.0	93.1	9.2	77.6	5.5	78.7	4.1	83.2	3.6	79.5	2.2
Sulfadoxine	97.4	1.2	93.6	5.1	90.8	7.3	75.9	3.2	92.2	10.5	77.5	3.9	84.5	5.8	89.1	3.0	86.8	2.2
Sulfisoxazole	96.4	1.6	92.0	4.4	87.6	7.9	82.4	4.7	101.6	9.0	86.4	4.4	77.1	8.7	79.6	2.6	79.3	1.6
Sulfabenzamide	91.9	3.0	88.7	3.0	86.0	11.0	73.6	8.9	91.4	6.8	77.2	4.4	69.5	8.5	71.7	1.8	70.1	3.7
Sulfaethoxypyridazine	101.2	2.6	94.8	4.2	90.4	8.5	75.8	5.5	93.2	7.8	77.4	3.9	79.9	6.4	83.6	2.4	80.5	1.6
Sulfaphenazole	102.5	1.2	92.7	4.9	88.5	8.3	74.7	3.9	97.1	8.7	80.8	4.8	79.0	10.0	82.8	3.8	77.9	4.5
Sulfaclozine	87.7	5.0	85.2	6.1	82.9	8.0	82.8	10.1	104.0	7.7	90.0	5.9	75.3	6.7	81.6	5.9	79.1	2.6
Sulfadimethoxine	97.1	1.9	94.1	4.4	89.3	7.2	76.9	4.3	92.6	8.5	77.5	3.9	78.3	6.5	81.1	3.3	77.6	1.3
Sulfabenz	100.4	5.5	92.1	4.8	88.4	7.6	90.1	4.8	98.8	6.2	84.0	3.6	116.3	9.6	94.3	10.3	80.7	6.1
Sulfapyrazole	97.8	1.2	92.5	5.2	88.1	9.9	76.9	4.6	93.5	9.8	78.3	3.9	71.5	6.4	73.7	3.0	72.7	2.9
Sulfaquinoxaline	87.0	2.6	87.8	6.9	84.3	8.1	88.8	2.8	107.9	7.1	93.3	4.2	70.4	5.4	73.0	4.9	70.9	1.4
Sulfanitran	116.4	8.0	97.9	9.0	93.5	9.3	72.8	13.8	98.8	13.6	82.2	13.4	78.0	8.5	81.9	5.8	83.5	6.0
Sulfasalazine	99.9	7.3	112.7	10.2	96.2	8.9	99.8	8.2	108.7	5.8	92.3	3.9	95.8	13.6	80.4	13.2	71.0	12.4
Tetracycline drugs																		
Tetracycline	78.2	3.5	86.1	1.4	85.5	8.1	71.5	5.8	85.7	7.4	73.1	4.7	71.4	3.7	77.0	7.0	74.5	3.8
Oxytetracycline	94.7	1.7	92.1	4.9	91.3	8.8	68.9	7.0	81.2	6.3	68.0	5.1	71.1	3.4	74.2	6.7	70.3	3.8
Chlortetracycline	96.0	2.7	90.3	6.1	90.6	8.1	65.1	11.1	77.5	10.4	67.5	5.9	71.9	5.5	74.5	2.6	73.6	1.5
Doxycycline	85.4	3.1	92.1	3.4	92.3	9.6	66.4	7.9	86.5	8.9	70.0	6.8	62.8	7.2	69.1	5.3	70.6	3.4

Rec.： recovery； /： no value.

### 2.5 实际样品测定

利用所建方法对15份水产品样品（包括5份草鱼肉、3份虾仁、2份扇贝丁和5份鲅鱼肉）进行检测。将检测结果与数据库中的磺胺类和四环素类药物信息进行比对，以实现非靶向筛查。实验发现，仅有1批次草鱼样品检出磺胺二甲基嘧啶，其余样品均为阴性。比对结果显示，母离子*m/z*偏差<5×10^‒6^，保留时间偏差<5%，同位素丰度比差异<15%，二级质谱库匹配得分为94.7分，确证该草鱼样品中含有磺胺二甲基嘧啶，所获二级质谱图与标准谱库的镜像比对见图5。利用磺胺二甲基嘧啶的草鱼肉基质匹配标准曲线进行定量分析，结果表明，磺胺二甲基嘧啶的含量为22.5 μg/kg。

**图5:**
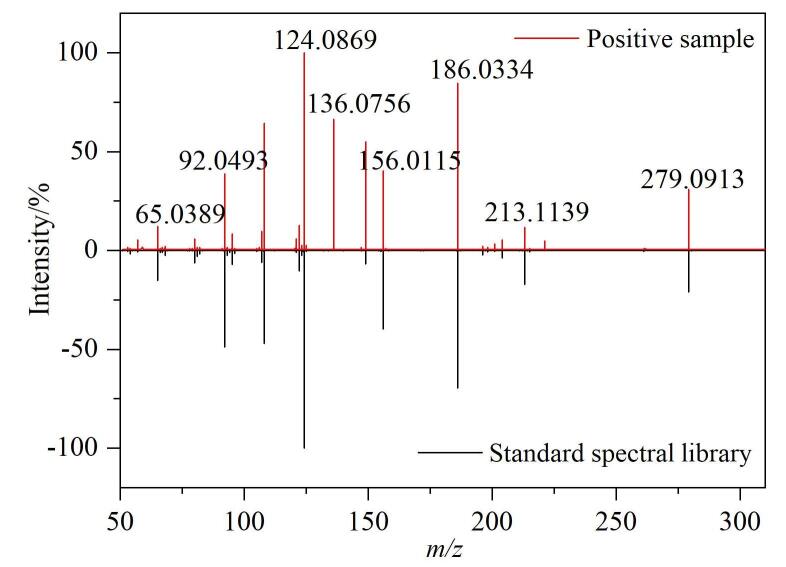
阳性样品中磺胺二甲基嘧啶的二级质谱图与标准谱库的镜像比对图

## 3 结论

本文将通过式固相萃取柱与超高效液相色谱-飞行时间质谱技术结合，建立了一种通用且可快速筛查和确证水产品中36种磺胺类和4种四环素类药物的分析方法。通过合理优化色谱分离条件，该方法在13 min内实现了7组共17种同分异构体的完全分离，对同分异构体的分离工作具有一定的借鉴意义。通过比较实验流程，选用PRiME HLB通过式固相萃取柱作为净化材料，在保证目标化合物回收率的前提下，简化了操作步骤，节省了样品前处理的时间。方法学验证和实际样品检测结果表明，该方法准确、稳定且通用性强，适用于水产品中磺胺类和四环素类药物的同时检测，可推广应用于食品检测领域。此外，结合本文所建立的数据库，该方法还可用于水产品样品中磺胺类和四环素类药物的非靶向筛查以及对疑似样品的再次确证。这有助于减少假阳性误判，实现磺胺类和四环素类药物的及时监测与准确研判。

## References

[1] Ye Q， Zhu Q M， Zhou F， et al. Chinese Journal of Chromatography， 2020， 38（7）： 868 34213296 10.3724/SP.J.1123.2020.02019

[2] Xie X， Huang S， Zheng J， et al. J Sep Sci， 2020， 43（9）： 1634 32043724 10.1002/jssc.201901341

[3] Sun D J . Feed Industry， 2023， 44（9）： 73

[4] Li A M， Huang Z， Lu W P， et al. Chinese Journal of Chromatography， 2014， 32（8）： 897 25434129

[5] Sun J， He T， Wang J P . Animal Husbandry & Veterinary Medicine， 2022， 54（4）： 142

[6] Wu Q， Liu X S， Dun X L， et al. Microchem J， 2021， 161： 105796

[7] Jorgensen M R， Bhurruth A Y， Bohov P . J Med Chem， 2009， 52： 1172 19175322 10.1021/jm801019s

[8] Yan Z M， Hu B Q， Li Q L， et al. J Chromatogr A， 2019， 1584： 33 30497825 10.1016/j.chroma.2018.11.039

[9] Shen R J， Huang L J， Liu R Q， et al. J Chromatogr A， 2021， 1655： 462518 34509690 10.1016/j.chroma.2021.462518

[10] GB 31650-2019

[11] GB 31650.1-2022

[12] Wu Q， Shabbir M， Peng D P， et al. Food Chem， 2021， 363： 130074 34120045 10.1016/j.foodchem.2021.130074

[13] Wu Q， Peng D P， Liu Q Y， et al. Front Microbiol， 2019， 10： 436 30915054 10.3389/fmicb.2019.00436 PMC6422943

[14] Chu J S， Xu Y， He Q H， et al. Journal of Food Science. 2011， 32（10）： 124

[15] Gu Y， Zhang S， Wang C J， et al. Journal of Food Safety and Quality， 2022， 13（3）： 993

[16] Chen Z G， Zhan C R， Guo P， et al. Journal of Food Science， 2007， 28（10）： 448

[17] Wang L， Zhang X S， Xu Z X， et al. Chinese Journal of Chromatography， 2002， 20（1）： 49 12541619

[18] Liang S H， Dai H R， Zhang H Y， et al. Chinese Journal of Chromatography， 2021， 39（6）： 624 34227323 10.3724/SP.J.1123.2020.12026 PMC9404201

[19] Liu P Y， Zhang H， Mi Z Y， et al. Chinese Journal of Chromatography， 2019， 37（10）： 1098 31642289 10.3724/SP.J.1123.2019.04005

[20] Sun H J， Li P W， Zhang B B， et al. Chinese Journal of Chromatography， 2022， 40（4）： 333 35362681 10.3724/SP.J.1123.2021.08010 PMC9404143

[21] GB/T 21316-2007

[22] GB/T 21317-2007

[23] GB 31656.1-2021

[24] Zhang N， Tang Z X， Wang L L， et al. Journal of Instrumental Analysis， 2023， 43（10）： 1343

[25] Kechagia M， Samanidou V， Kabir A， et al. J Sep Sci， 2018， 41（3）： 723 29150925 10.1002/jssc.201701205

[26] Yan Z H， Li X W， Xia X . Journal of Instrumental Analysis， 2023， 42（10）： 1309

[27] Shu D， Jiang M， Wu H， et al. Chinese Journal of Analysis Laboratory， 2024， 43（8）： 1139

[28] Xia B L， Wang S T， Yin J J， et al. Chinese Journal of Chromatography， 2023， 41（7）： 591 37387280 10.3724/SP.J.1123.2022.09008 PMC10311621

[29] Vartanian V H， Goolsby B， Brodbelt J S . J Am Soc Mass Spectrom， 1998， 9（10）： 1089

[30] Matuszewski B K， Constanzer M L， Chavezeng C M . Anal Chem， 2003， 75（13）： 3019 12964746 10.1021/ac020361s

